# Construction and Identification of a Breast Bioreactor for Human-Derived Hypoglycemic Protein Amylin

**DOI:** 10.3390/life14020191

**Published:** 2024-01-28

**Authors:** Kongwei Huang, Xiuying Yan, Zhipeng Li, Fuhang Liu, Kuiqing Cui, Qingyou Liu

**Affiliations:** 1 Guangdong Provincial Key Laboratory of Animal Molecular Design and Precise Breeding, School of Life Science and Engineering, Foshan University, Foshan 528225, China; yanxiuying@cibr.ac.cn (X.Y.); kqcui@fosu.edu.cn (K.C.); 2 State Key Laboratory for Conservation and Utilization of Subtropical Agro-Bioresources, Guangxi University, Nanning 530004, China; zp.li@gxu.edu.cn (Z.L.); 1708404002@st.gxu.edu.cn (F.L.)

**Keywords:** mammary gland bioreactor, Amylin, diabetes, metagenomics

## Abstract

The mammary gland of mammals can generate numerous bioactive proteins. To express the human amylin protein in the mammary glands of domestic animals, we engineered a transgenic mammary gland bioreactor. For this study, we produced transgenic mice through prokaryotic microinjection. RT-PCR, qPCR, and Western blotting confirmed the presence of transgenes in the mice. The ELISA assay indicated an amylin yield of approximately 1.44 μg/mL in the mice milk. Further research revealed that consuming milk containing amylin resulted in a slight, but insignificant enhancement in food consumption, blood sugar equilibrium, and glucose tolerance. The influence of amylin-fortified milk on the abundance of fecal strains in mice was examined, and a significant difference in the quantity of strains needed for fatty acid synthesis and metabolism was discovered. The amylin protein gathered from humans is safe to consume, as no harmful effects were detected in the mice. Our study examined the production of human amylin using a new safety strategy that could potentially alleviate diabetic symptoms in the future through oral administration of milk containing amylin.

## 1. Introduction

In 2021, the International Diabetes Federation (IDF) and the Food and Agriculture Organization (FAO) reported a worldwide total of 7.9 billion individuals with diabetes and over 1 billion people considered obese. Clinical interventions for diabetes management encompass utilizing oral or injected drugs that promote insulin secretion, reduce hepatic glycogen decomposition, and enhance hormone receptor sensitivity [1,2,3]. Conversely, obesity chiefly obtains treatment for weight reduction through active dieting and surgery. Metformin and liraglutide are noted treatments for both diabetes and weight loss. Additionally, amylin has been shown to be effective in treating diabetes and for weight control [4,5,6]. Amylin, also known as islet amyloid polypeptide (IAPP), is a neuroendocrine hormone made up of 37 amino acids that is co-expressed and co-secreted with insulin in pancreatic β-cells [7]. β-cell death is a crucial component of type 2 diabetes, and the misfolding of IAPP is considered one of the causative factors [8]. Insulin is known to play a crucial role in reducing postprandial blood glucose levels, while glucagon maintains fasting blood glucose by increasing gluconeogenesis [9,10]. Amylin, specifically, has been shown to regulate both insulin and glucagon [11]. Additionally, amylin can also inhibit glucagon secretion. High concentrations of amylin can inhibit insulin secretion, while low concentrations of amylin promote insulin secretion in a dose-dependent manner [12]. Not only does amylin have the ability to negatively regulate glucagon secretion, but it also works on the central nervous system to maintain energy balance and induce feelings of fullness [13,14]. In diabetic patients, postprandial blood glucose levels increase rapidly due to insulin insensitivity or inadequacy, which can be regulated by amylin [15]. Amylin induces abdominal fullness by stimulating the nerve center [11]. On one hand, it slows down gastric digestion to prevent the swift delivery of large amounts of glucose into the bloodstream. On the other hand, it sends a satiety signal to the brain, which suppresses the craving for food. This results in decreased food intake in individuals with type 2 diabetes, thereby promoting weight management [16,17]. Incorrectly folded and sheared amylin can result in amyloid deposition in a β-sheet structure [18,19,20], which may lead to the loss of positive roles and potentially result in cellular apoptosis and oxidative stress [21,22]. Thus, certain amino acids in amylin were replaced to reduce aggregation. However, the currently high drug prices are attributed to the complex production process [23,24,25].

Diverse expression systems have been developed to obtain protein drugs. Escherichia coli, yeast, insects, plants, and mammals have been utilized to produce a wide array of recombinant proteins [26,27,28,29]. In 1987, the production of recombinant protein drugs from transgenic animals brought about a new era which culminated in 2006 with the introduction of human antithrombin III. The utilization of mammary gland bioreactors for the production of exogenous active proteins has received considerable attention due to its numerous advantages. Animal mammary glands’ recombinant proteins possess high bioactivity and complete post-translational modifications of proteins [30]. Moreover, the protein is secreted via milk excretion, and the protein in milk undergoes a complete separation and purification production process [31,32]. The mammary gland has a substantial capability to process foreign proteins and provides a high yield in an environmentally friendly and cost-effective manner. Numerous proteins, including human lactoferrin, serum albumin, and interferon, have been effectively expressed in the mammary gland with transgenic animals, thus promoting protein drug development [33,34,35]. The injection of human amylin has previously been demonstrated to enhance symptom relief in diabetic mice [36]. The small peptide present in milk can be absorbed directly into the bloodstream via the small intestine. This could potentially aid in improving diabetic symptoms by consuming milk containing amylin of only 3.2 kDa in size.

In this study, the amylin mature peptide gene sequence was selected to construct mammary gland-specific expression vectors. The genetically modified human amylin protein was then expressed in the mammary glands of mice. The study continuously recorded the growth and metabolism data of the offspring consuming transgenic milk for the purpose of evaluating the function of the amylin protein. This research offers a valuable reference for the production of amylin within the mammary gland and its potential use in treating diabetes and controlling weight.

## 2. Materials and Methods

### 2.1. Construction of Transgene Cassette

Human Amylin gene (hAmylin) sequence was obtained from Genebank (ID: NM_000415.2) and optimized using mammalian preference codons online (https://jcat.de/). The optimized sequence was synthesized by Sangon Biotech company (Shanghai, China) with Xho I restriction sites at both ends (Table 1) and inserted into pBC1 vector (Invitrogen, Carlsbad, CA, USA). Meanwhile, we also constructed a reporter plasmid containing EGFP to build a cell expression model.

### 2.2. The Expression of Amylin in Bcap37 Cell Model

The pBC1-Amylin-GFP vector was transfected into Bcap37 cells at about 80% cell density. The cell electro-transfection were proceed using 225 V for 10 ms with 2 mm gap shock cups. The fluorescence microscope (NIKON, Tokyo, Japan) was used to observe the fluorescent signal. Total RNA of cells were isolated using Trizol solution (Invitrogen, Carlsbad, CA, USA) and First-strand cDNA was synthesized using RevertAid First Strand cDNA Synthesis Kit (K1622, Thermo Fisher Scientific, Waltham, MA, USA). Primers were designed using oligo 7 software (Table 2) and synthesized in Sangon Biotech company (Shanghai, China). The expressions level were quantified using a SYBR Green based real-time PCR kit (FastStart Universal SYBR Green Master, Roche, Basel, Switzerland) according to the manufacturer’s instruction. In brief, a 20 μL mixture was performed in each run as follows: 100 ng cDNA, 8 μL H_2_O, 10 μL Faststart Universal SYBR Green Master (ROX), and 0.5 μM aliquots of both forward and reverse primers. The thermal cycling profile started with a 3-min dwell temperature of 95 °C, followed by 40 cycles of 30 s at 95 °C, 40 s at 60 °C, and a final step during which fluorescence was acquired. After 40 cycles, a melting curve was generated by temperature increments of 0.1 °C starting three 3 times, and relative gene expression was calculated using the 2^−ΔΔCt^ method with β-actin as the reference gene.

### 2.3. Generating and Identification of Transgenic Mice

The pBC1-hAmylin vector was digested with restriction endonuclease Not I and Sal I to remove the prokaryotic sequence, and the remaining fragment was purified with OMEGA Gel Extraction Kit (Omega Bio-Tek, Norcross, GA, USA). Then, it was injected into FVB/NJ strain mice zygotes according to standard protocols to generate transgenic mouse [35]. The transgenic mice and its offspring were identified by genomic PCR with DNA samples extracted from tail biopsy. The founder transgenic mice were obtained and the homozygous transgenic mice were obtained by inbreeding screening.

### 2.4. Expression Analysis of Amylin

The screened transgenic mice were sacrificed and the total RNA of diverse tissues of the transgenic mice, including heart, liver, spleen, lung, kidney, small intestine, muscle, pancreas, breast, ovarian and testis, were extracted with Trizol solution (Invitrogen, Carlsbad, CA, USA) and reverse transcribed to cDNA with the RevertAid First Strand cDNA Synthesis Kit (K1622, Thermo Fisher Scientific, Waltham, MA, USA). The specific expression of human amylin in the mice were detected using taqman probe (Table 2) based qPCR. The expression of human amylin in the milk of transgenic mice was further analyzed by western blot. The milk was collected with a milk collection machine (Automated Experimental Milker WAT-2006, Tokyo, Japan) and diluted with 3 volumes of ddH_2_O. It was then centrifuged at 8000 rpm with 4 °C for 20 min and the middle layer was collected for further use of western blot. To obtain the protein of breast tissue, tissues were ground in liquid nitrogen and lysed with RIPA and PMSF (Solarbio, Beijing, China). For western blot analysis, protein samples were separated using a 12% SDS-PAGE (TGX Stain-Free™ FastCast™ Acrylamide Solutions, Bio-Rad, Hercules, CA, USA) and transferred to 0.22 μm nitrocellulose membrane (Pall Life Sciences, New York, NY, USA). The membrane were blocked with 5% skimmed milk (BD Biosciences, San Jose, CA, USA) at room temperature for 2 h and then incubated with anti-amylin monoclonal antibody (1:700, ab103580, Abcam, Cambridge, UK) and anti-Actin polyclonalantibody (1:5000, 20536-1-AP, Proteintech, Chicago, IL, USA) overnight at 4 °C respectively. After that, the membranes were incubated with HRP conjugated second antibody (1:10,000, SA00001-2, Proteintech, Chicago, IL, USA) at room temperature for 2 h. The hybridization signal was finally interacted with ECL detection reagents and the band signals were captured by the imaging system. (Gel Doc TM XR+, Bio-Rad, Hercules, CA, USA). The concentration of human amylin in the milk of transgenic mice was determined with an ELISA kit (KB10399, Jianglaibio, Shanghai, China) according to the protocols. Mice milk were diluted 10-fold before testing and the 450 nm absorbance was recorded by microplate reader (Infinite M200 PRO, TECAN, Männedorf, Switzerland). The expression level of the human amylin in the transgenic mice was quantified by comparing with the standard curve and each sample was repeated 5 times.

### 2.5. Biological Activity of Amylin from Mice Milk

Wild-type newborn mice were divided into two groups (*n* = 5) and fed by transgenic mouse milk or WT mouse milk, respectively. The body weight, body temperature, food intake, water intake, and random blood glucose of the mice were continuously recorded until 8 months of age. The glucose tolerance test (GTT) was performed as follow: Mice in two groups were fasted overnight and then oral administration with 10% D-glucose solution (1 g/kg body weight). The blood glucose was recorded at 0 h, 0.5 h, 1 h, 1.5 h, 2 h, 2.5 h, and 3 h after the administration. The blood glucose curve and the area under the curve was calculated.

### 2.6. Intestinal Metagenomics Analysis

Studies have found that the intestinal microbial abundance can be affected by glycolipid metabolism [37,38]. Therefore, intestinal metagenomics of mice that feed by transgenic amylin milk were studied to identify the effect of glycolipid metabolism regulated by amylin. In brif, fecal samples of two groups of mice (6 months after lactation, *n* = 3) were collected at 8:00 a.m., 2:00 p.m. and 10:00 p.m. and mixed for intestinal microbiome analysis. Raw data were quality controlled using Trimmomatic (v.0.39) software, and then the host genome (GCF_000001635 GRCm39 mus genomic) was removed by Bowtie2 (v2.3.5.1) software [39]. Clean data species and abundance were identified by MetaPhlAn2 (v2.7.7). According to the Kruskal-Wallis test and pairwise Wilcoxon test, *p* value ≥ 0.05 are not further analyzed. LDA and LEfSe were used to identified each microbial feature with differential abundance [40]. It was considered that the LDA score > 2 and *p* value < 0.05 as different taxa. Heat map construction using R (v 3.6.1).

### 2.7. Statistical Analyses

All tests were performed using GraphPad Prism (v6.0) (GraphPad Software, Inc., San Diego, CA, USA), SPSS Statistics (V. 24.0.0.0) (SPSS Inc., Chicago, IL, USA) or R software (version 3.6.1). *p*-values < 0.05 were considered significant.

## 3. Results

### 3.1. Vector Construction and Expression in Bcap37 Cells

**Figure 1:**
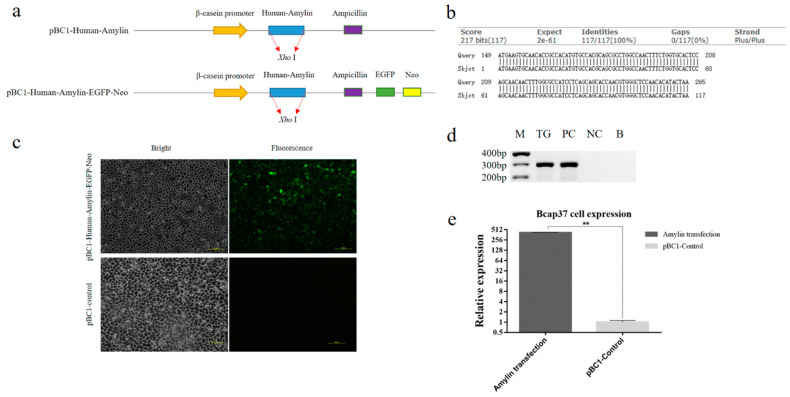
Plasmid structure and identification. (**a**) Schematic diagram of the constitution of the pBC1-hAmylin plasmid. Vector with EGFP was used to detect the expression of amylin in Bcap37 cell. (**b**) Blast result of the Sanger sequencing of amylin. (**c**) Fluorescence detection of the expression of pBC1-hAmylin-EGFP plasmid in Bcap37 cells. (**d**) RT-PCR detection of amylin in Bcap37 cells. TG indicates Bcap37 cells transfected with pBC1-Human-Amylin-EGFP plasmid; PC indicates positive control; NC indicates negative control (Bcap37 cells without transfection); B indicates the blank control. (**e**) qPCR detection of human amylin in Bcap37 cells. The data in the figure is the mean ± SE; **, *p* < 0.01.

The optimized human amylin sequence was inserted into the *Xho* I site of pBC1 vector and GFP was further inserted to investigate the expression in cell model (Figure 1a). Sanger sequencing and blast alignment showed that human amylin gene were correctly inserted into the pBC1 vector (Figure 1b). To confirm the function of the constructed vector, the pBC1-Amylin-EGFP plasmid was transfected into Bcap37 cells. Fluorescence detection was performed 24 h after the transfection, and green fluorescence was observed in the transfected-cells with no observed fluorescence in the non-transfected cells (Figure 1c). Further reverse transcription PCR verified the expression of amylin (Figure 1d) and qPCR results showed that the expression of amylin in transfected cells were significantly higher than that of control cells (Figure 1e). Results suggested that the constructed vector can be used in the expression of human amylin in mammal breast cells.

### 3.2. Screen of Transgenic Mice and Expression Analysis of Amylin

**Figure 2:**
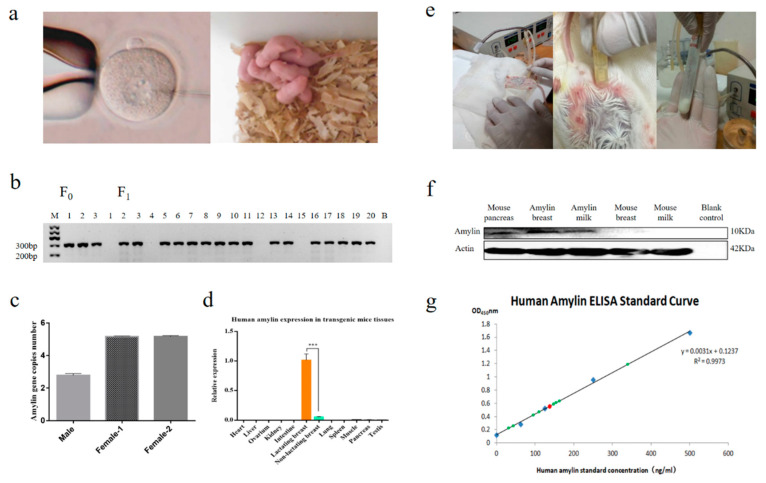
Generation and screen of human amylin transgenic mice. (**a**) Production of transgenic mice by microinjection. (**b**) Screen of the genetically modified mice by PCR, B indicates blank control. (**c**) PCR identification of transgenic fragments of mouse genomic DNA. In the figure, F_0_ represents the first generation of transgenic mice, two females and one male, and F_1_ represents the second generation of transgenic mice, the offspring produced by mating with the F_0_ generation of transgenic mice. The mice with bands in the figure are transgenic mice and those without bands are non-transgenic mice. (**d**) Detection of human amylin in diverse tissues of transgenic mice by qPCR The orange column represents lactating breast tissue, while the green column represents non-lactating breast tissue (*n* = 5). ***, *p* < 0.001. The data in the figure is the mean ± SE. (**e**) Collection of the milk of mice using the Automated Experimental Milker WAT-2006. (**f**) Detection of the expression of human amylin in the milk by western blot. (**g**) Detection of the yield of human amylin in the milk by ELISA. The graph displays standard curves constructed from amylin protein standards in blue points. The green points represent the concentration of amylin protein in the milk produced by each transgenic mouse, while the red points represent the average concentration of amylin protein in the milk produced by all transgenic mice.

The founder mice were obtained after 3 weeks of pregnancy (Figure 2a) and tail DNA was used to screen the insertion of exogenous gene through PCR (Figure 2b). The transgenic copy number of the 3 founder mice was detected and result showed that 3/5/5 amylin gene copies were found in the genome, respectively (Figure 2c). The human amylin specific Taqman probe based qPCR analysis showed that amylin was only expressed in the breast tissue of lactating mice (Figure 2d). The milk of transgenic mice and wild type mice were collected (Figure 2e) and the expression of amylin was further identified using western blot and ELISA analysis. The results of western blot showed that human amylin was expressed in the breast and milk of transgenic mice with no expression in the wild-type mice (Figure 2f). ELISA assay showed that the expression of amylin protein in transgenic mouse milk was 1.44 ± 0.3 mg/mL (*n* = 5) (Figure 2g).

### 3.3. Function Assessment of the Amylin in Transgenic Milk

**Figure 3:**
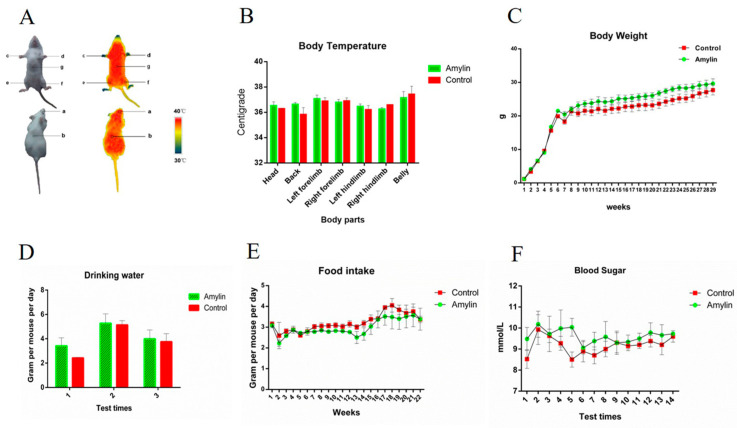
Analysis of basic indexes of energy metabolism in mice. The amylin group indicates mice feed with amylin milk from transgenic mice (*n* = 4), the control group indicates mice feed with milk from wild-type mice (*n* = 4). (**A**) Schematic diagram of the detection of body temperature. (**B**) Statistical analysis of the body temperature. (**C**) The weight of mice was recorded once a week. (**D**) Statistical analysis of the water intake of mice. (**E**) Statistical analysis of food intake of mice. (**F**) Statistical analysis of the blood glucose concentration of mice. The data in the figures are the mean ± SE.

**Figure 4:**
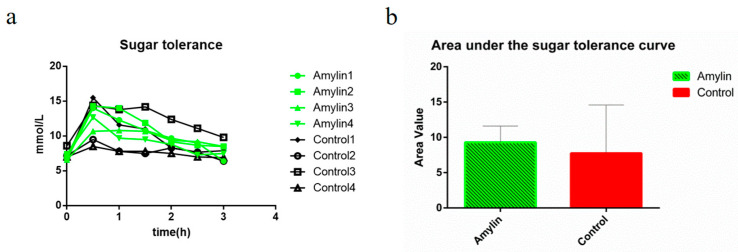
Oral glucose tolerance test (OGTT) in mice. (**a**) Trend plots of blood glucose concentrations over time after OGTT experiments. (**b**) Statistical analysis of the area under the glucose tolerance curve. The data in the figure is the mean ± SE.

In this study, the expression of human amylin can be continuously detected in the milk of mice until the eighth generation and the function of this milk derived amylin was further investigated. As one of the direct manifestations of the energy metabolism, body temperature of the mice were detected and no significant different was found between mice feed by amylin milk and wild type milk (Figure 3A,B). Amylin intake resulted in a small and non-significant improvement in body weight (Figure 3C) and water intake (Figure 3D), while the food intake was slightly suppressed (Figure 3E). Moreover, the blood glucose in the mice feed with amylin milk were slightly higher and less fluctuation than that in control group (Figure 3F). The oral glucose tolerance test was also performed and amylin intake improved the glucose tolerance, though no significant different was found comparing with control group (Figure 4a,b). These results suggested that, though without significant different, intake of this amylin milk improved the glycometabolism of mice, and also indicated that this transgenic milk is safety as no significant negative effect on the health of mice.

### 3.4. Intestinal Microbiome Analysis

**Figure 5:**
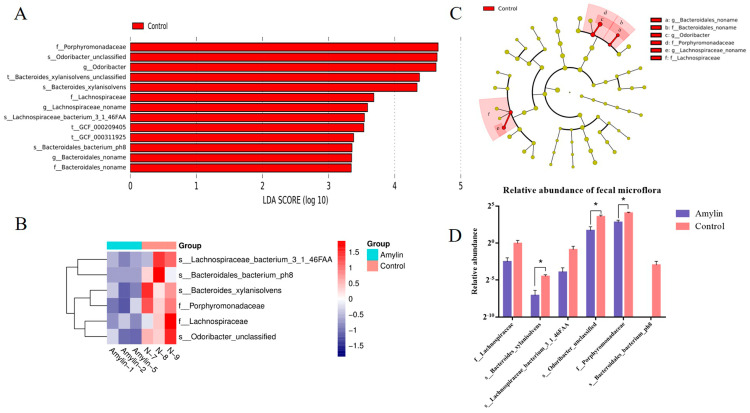
Effect on the abundance of intestinal flora in mice. (**A**) Results of Linear discriminant analysis (LDA) score from Lefes analysis. Compared with the control mice, three families, three genera and four species level flora were significantly down regulated in the mice of the amylin group. (**B**) Cluster heatmap of key differential flora with red representing high abundance and purple representing low abundance. (**C**) The key differences between the two groups in terms of flora using the *t*-test revealed significant differences (*p* < 0.05) at the two species level and one genus level. (**D**) Taxonomic results of the differential flora of the two groups of gut microbes, which can be divided into three taxa. The data in the figure is the mean ± SE; *, *p* < 0.05.

Based on the role of amylin in glucose metabolism and gastric emptying, we further analyzed the effect of drinking this amylin milk on the fecal strain abundance in mice. Results showed that, comparing to the control group, significant difference was found in the abundance of strains that crucial in fatty acid synthesis and metabolism. linear discriminant analysis effect size (LEfSe) analysis showed strains in three families, three genera and four species were down-regulated in the amylin milk intake group (Figure 5A). Cluster analysis showed that the abundance of strains in two family levels (Lachnospiraceae and Porphyromonadaceae) and four species levels (Bacteroides_Xylanisolvens, Lachnospiraceae_Bacterium_3_1_46FAA, Odoribacter_Unclassified and Bacteroidales_Bacterium_Ph8) were significantly decreased in mice feed by amylin milk (Figure 5B). Further analysis found that the Bacteroides, lachnospiraceae and porphyromonadaceae in the amylin intake group by taxonomic analysis, the differential flora could be classified into three categories (Figure 5C) and their abundances were significantly down-regulated than those in the control group (Figure 5D). These changes of strains may affect the digestion, absorption and energy metabolism of the host and finally beneficial to the improvement of diabetes and obesity.

## 4. Discussion

Amylin is known as a neuroendocrine peptide hormone with anorectic and gluco-regulatory actions. In this study, we provided a transgenic mouse model which can secrete human amylin specifically in the mammary gland and analyzed the function of this milk derived human amylin. We showed that intake of this milk derived amylin resulted in a small and non-significant improvement in food intake, blood sugar balance and glucose tolerance. It is interesting to notice that, though the food intake in mice feed by amylin milk was decreased, the body weight increment was slightly higher than control group, indicating that amylin can improve the energy utilization efficiency of organism. Further intestinal microbiome analysis showed that the abundance of strains that crucial in fatty acid synthesis and metabolism were significantly down regulated, providing novel understanding about the potential effect mechanism of amylin on health.

Currently, diverse expression system have been used in the production of human amylin protein, while no procedure has been described to obtain high yield of native amylin protein. As the most commonly used protein expression system, prokaryotic expression system is known for its practical, and is characterized by its broad applicability and easy realization. It has been used to produce human amylin and obtained about 2.5 mg/L amylin protein in *E. coli* [41]. However, it is limited by the ability of post-transcriptional modification in prokaryotes to modify protein, which may finally affected biological activity of the protein. Amylin can also be obtained from mammalian cells, however, the cell expression system are high cost with low protein yield and difficult to satisfy the needs in actual production [42]. At present, animal breast bioreactor has been used to express diverse medicinal proteins, including lactoferrin, interferon, serum albumin, and the benefit of this systems lies in the high level and quality of the product obtained at a relatively low cost with native biological activity [30,33,34,35]. In this study, human amylin protein were obtained from the milk of transgenic mice with native biological activity and high yield, which provide a potential candidate procedure to produce medicinal human amylin. Though heterotopic expression of foreign proteins is frequently found in animal bioreactor, strictly specific expression of human amylin in the breast was observed in our mouse model [43,44]. Many studies have shown that the expression of inserted genes can be controlled though specific promotor and the specific expression in our study may own to the use of lactation mammal gland specific casein promotor [45,46].

Function and safety was the most worthy issue to concern, whether for the obtained protein, or for the health of genetically modified mice. As we know, the main function of amylin was the regulation of blood glucose. Previous studies have shown that injection of amylin reduced postprandial blood glucose levels in patients with type 2 diabetes and even cause severe hypoglycemia when combined with insulin [15]. Intake of milk derived amylin also caused slightly glucose decreases in this study, though with no significant difference. Though high yield of amylin was found in the milk, the mice were free choice feeding and the dose of amylin intake is unsure. Therefore, the dose-dependent effect may led to the insignificant role of amylin in this study. When it comes to the health of the genetically modified mice, by continuously recording of the body weight, diet, and body temperature of the mice, no significant difference was found between the transgenic and wild type mice, indicating that this is a viable candidate method to produce the human amylin protein.

Diverse studies have found that the abundance of intestinal microflora is important to human health disorder of the intestinal flora may lead to serious diseases [47,48,49]. Intestinal microbiological disorders in obese and diabetic patients can lead to the reduction of short chain fatty acid synthesis [50]. In this study, we found that the relative abundance of porphyromonadaceae, lachnospiraceae and odoribacter were significantly decreased in mice fed by amylin milk. The abundance of porphyromonadaceae in fecal samples is significantly decreased in obese mice, which is consist with our study [51]. In addition, researchers have found that lachnospiraceae is an important strain of short chain fatty acid metabolism, and is a microbial marker in maternal and infant obesity genetics [50]. Children with a large number of lachnospiraceae have a 5-fold increase in the probability of overweight at one year old [49]. Interestingly, the abundance of lachnospiraceae in the feces of mice fed by amylin milk was lower than that in normal mice. Moreover, the abundance of odoribacter was also decreased in the amylin group. Odoribacter has been found significantly increased in the intestine of mice fed with high fat diet, which regulated the expression of glycolysis-related genes (e.g., Pparg), and may serve as a potential target for the treatment of obesity [48]. In this study, decreased abundance of porphyromonadaceae, lachnospiraceae and odoribacter were observed in the feces of mice fed with amylin milk. These evidences suggested that intake of the milk containing amylin protein might reduce the risk of obesity by regulating the abundance of intestinal microflora.

## 5. Conclusions

This artical presents the novel expression of native human amylin mature protein in milk from transgenic mice. Consumption of this milk-derived amylin led to a non-significant yet minor improvement in food intake, glucose tolerance, and blood sugar balance. Furthermore, the safety of the human amylin protein was confirmed through the absence of significant adverse effects in mice. Although this milk-derived human amylin is insufficient for clinical treatment purposes, it presents a novel strategy and reference point for future production of amylin in livestock milk as well as enhanced comprehension of amylin’s actions.

## Figures and Tables

**Table 1:** Amylin improved sequence and mature peptide sequence.

Amylin Gene	Sequence
Improved sequence	CTCGAGATGAAGTGCAACACCGCCACATGTGCCACGCAGCGCCTGGCCAACTTTCTGGTGCACTCCAGCAACAACTTTGGCGCCATCCTCAGCAGCACCAACGTGGGCTCCAACACATACTAACTCGAG

Mature peptide	MKCNTATCATQRLANFLVHSSNNFGAILSSTNVGSNTY *

The asterisk represents the position of the protein termination codon. Letters with underline indicate restriction enzyme *Xho* Ⅰ site.

**Table 2:** Primers used in this study.

Primers Name	Sequence
pBC1-Universal	F: 500B4-ATTGACAAGTAATACGCTGTTTCCTC-3′
	R: 5′-ATCAGAAGTTAAACAGCACAGTTAG-3′
Human-amylin	F: 5′-ATGAAGGTGCTGATCCTGGCC-3′
	R: 5′-AGTAGGTGTTGCTGCCCACG-3′
Human-amylin-probe-T	5′FAM-CCTGGTGGCCCTGGCCATCGC-NFQ-MGB 3′
Mus-actin	F: 5′-CGATGCCCTGAGGCTCTTT-3′
	R: 5′-TGGATGCCACAGGATTCCA-3′
Mus-actin-probe-T	5′HEX-CCAGCCTTCCTTCTT-NFQ-MGB 3′

F indicates forward primer and R indicates reverse primer. FAM and HEX are reporters used at the 5′ end of Taq man probe, and NFQ-MGB is quencher at the 3′ end. The final concentration of primer used is 10 μM, and probe is 30 μM.

## Data Availability

All of the dates generated and analyzed during this study are included in this paper. Metagenomic raw data have been uploaded to NCBI and can be accessed through BioProject ID: PRJNA694540. Additional datasets used and/or analyzed during the current study are available from the corresponding author on reasonable request.

## References

[1] Harris S., Abrahamson M., Ceriello A., Charpentier G., Evans M., Lehmann R., Liebl A., Linjawi S., Holt R., Hosszúfalusi N. (2020). Clinical Considerations When Initiating and Titrating Insulin Degludec/Liraglutide (IDegLira) in People with Type 2 Diabetes. Drugs.

[2] Zinman B., Ahrén B., Neubacher D., Patel S., Woerle H., Johansen O. (2016). Efficacy and Cardiovascular Safety of Linagliptin as an Add-On to Insulin in Type 2 Diabetes: A Pooled Comprehensive Post Hoc Analysis. Can. J. Diabetes.

[3] Goldenberg R., Steen O. (2019). Semaglutide: Review and Place in Therapy for Adults with Type 2 Diabetes. Can. J. Diabetes.

[4] Baggio L., Drucker D. (2020). Glucagon-like peptide-1 receptor co-agonists for treating metabolic disease. Mol. Metab..

[5] Meleleo D., Cibelli G., Valenzano A., Mastrodonato M., Mallamaci R. (2023). The Effect of Calcium Ions on hIAPP Channel Activity: Possible Implications in T2DM. Membranes.

[6] Ghusn W., Hurtado M.D., Acosta A. (2022). Weight-centric treatment of type 2 diabetes mellitus. Obes Pillars.

[7] Cooper G., Day A., Willis A., Roberts A., Reid K., Leighton B. (1989). Amylin and the amylin gene: Structure, function and relationship to islet amyloid and to diabetes mellitus. Biochim. Biophys. Acta.

[8] Chaari A., Abdellatif B., Nabi F., Khan R. (2020). Date palm (*Phoenix dactylifera* L.) fruit’s polyphenols as potential inhibitors for human amylin fibril formation and toxicity in type 2 diabetes. Int. J. Biol. Macromol..

[9] Vergari E., Knudsen J., Ramracheya R., Salehi A., Zhang Q., Adam J., Asterholm I., Benrick A., Briant L., Chibalina M. (2019). Insulin inhibits glucagon release by SGLT2-induced stimulation of somatostatin secretion. Nat. Commun..

[10] Tura A., Pacini G., Yamada Y., Seino Y., Ahrén B. (2019). Glucagon and insulin secretion, insulin clearance, and fasting glucose in GIP receptor and GLP-1 receptor knockout mice. Am. J. Physiol. Regul. Integr. Comp. Physiol..

[11] Ling W., Huang Y., Qiao Y., Zhang X., Zhao H. (2019). Human Amylin: From Pathology to Physiology and Pharmacology. Curr. Protein Pept. Sci..

[12] Akesson B., Panagiotidis G., Westermark P., Lundquist I. (2003). Islet amyloid polypeptide inhibits glucagon release and exerts a dual action on insulin release from isolated islets. Regul. Pept..

[13] Reiner D., Mietlicki-Baase E., Olivos D., McGrath L., Zimmer D., Koch-Laskowski K., Krawczyk J., Turner C., Noble E., Hahn J. (2017). Amylin Acts in the Lateral Dorsal Tegmental Nucleus to Regulate Energy Balance Through Gamma-Aminobutyric Acid Signaling. Biol. Psychiatry.

[14] Mietlicki-Baase E., Hayes M. (2014). Amylin activates distributed CNS nuclei to control energy balance. Physiol. Behav..

[15] Hieronymus L., Griffin S. (2015). Role of Amylin in Type 1 and Type 2 Diabetes. Diabetes Educ..

[16] Woods S., Lutz T., Geary N., Langhans W. (2006). Pancreatic signals controlling food intake; insulin, glucagon and amylin. Philos. Trans. R. Soc. Lond. Ser. B Biol. Sci..

[17] Apovian C., Okemah J., O’Neil P. (2019). Body Weight Considerations in the Management of Type 2 Diabetes. Adv. Ther..

[18] Schultz N., Byman E., Fex M., Wennström M. (2017). Amylin alters human brain pericyte viability and NG2 expression. J. Cereb. Blood Flow Metab. Off. J. Int. Soc. Cereb. Blood Flow Metab..

[19] Liu M., Hoskins A., Verma N., Bers D., Despa S., Despa F. (2018). Amylin and diabetic cardiomyopathy—Amylin-induced sarcolemmal Ca leak is independent of diabetic remodeling of myocardium. Biochim. Biophys. Acta. Mol. Basis Dis..

[20] Ren B., Liu Y., Zhang Y., Cai Y., Gong X., Chang Y., Xu L., Zheng J. (2018). Genistein: A Dual Inhibitor of Both Amyloid β and Human Islet Amylin Peptides. ACS Chem. Neurosci..

[21] Singh S., Bhowmick D., Pany S., Joe M., Zaghlula N., Jeremic A. (2018). Apoptosis signal regulating kinase-1 and NADPH oxidase mediate human amylin evoked redox stress and apoptosis in pancreatic beta-cells. Biochim. Biophys. Acta Biomembr..

[22] Press M., Jung T., König J., Grune T., Höhn A. (2019). Protein aggregates and proteostasis in aging: Amylin and β-cell function. Mech. Ageing Dev..

[23] Smaoui M., Waldispühl J. (2015). Complete characterization of the mutation landscape reveals the effect on amylin stability and amyloidogenicity. Proteins.

[24] Paul A., Kalita S., Kalita S., Sukumar P., Mandal B. (2017). Disaggregation of Amylin Aggregate by Novel Conformationally Restricted Aminobenzoic Acid containing α/β and α/γ Hybrid Peptidomimetics. Sci. Rep..

[25] da Silva D., Fontes G., Erthal L., Lima L. (2016). Amyloidogenesis of the amylin analogue pramlintide. Biophys. Chem..

[26] Hayat S., Farahani N., Golichenari B., Sahebkar A. (2018). Recombinant Protein Expression in *Escherichia coli* (*E. coli*): What We Need to Know. Curr. Pharm. Des..

[27] Suzuki R., Sakakura M., Mori M., Fujii M., Akashi S., Takahashi H. (2018). Methyl-selective isotope labeling using α-ketoisovalerate for the yeast Pichia pastoris recombinant protein expression system. J. Biomol. NMR.

[28] Chang J., Lee S., Kim J., Wang C., Nai Y. (2018). Transient Expression of Foreign Genes in Insect Cells (sf9) for Protein Functional Assay. J. Vis. Exp. JoVE.

[29] Jazayeri S., Amiri-Yekta A., Bahrami S., Gourabi H., Sanati M., Khorramizadeh M. (2018). Vector and Cell Line Engineering Technologies Toward Recombinant Protein Expression in Mammalian Cell Lines. Appl. Biochem. Biotechnol..

[30] Li Z., Cui K., Wang H., Liu F., Huang K., Duan Z., Wang F., Shi D., Liu Q. (2019). A milk-based self-assemble rotavirus VP6-ferritin nanoparticle vaccine elicited protection against the viral infection. J. Nanobiotechnol..

[31] Mohsin A., Sukor R., Selamat J., Meor Hussin A., Ismail I., Jambari N., Jonet A. (2020). A highly selective two-way purification method using liquid chromatography for isolating α-casein from goat milk of five different breeds. J. Chromatogr. B Anal. Technol. Biomed. Life Sci..

[32] Wang X., Ma T., Yu H., Chen Z., Zhu B., Chen W., Sun S., Li Z. (2020). Purification of sialoglycoproteins from bovine milk using serotonin-functionalized magnetic particles and their application against influenza A virus. Food Funct..

[33] Wang M., Sun Z., Yu T., Ding F., Li L., Wang X., Fu M., Wang H., Huang J., Li N. (2017). Large-scale production of recombinant human lactoferrin from high-expression, marker-free transgenic cloned cows. Sci. Rep..

[34] Luo Y., Wang Y., Liu J., Lan H., Shao M., Yu Y., Quan F., Zhang Y. (2015). Production of transgenic cattle highly expressing human serum albumin in milk by phiC31 integrase-mediated gene delivery. Transgenic Res..

[35] Li H., Liu Q., Cui K., Liu J., Ren Y., Shi D. (2013). Expression of biologically active human interferon alpha 2b in the milk of transgenic mice. Transgenic Res..

[36] Zhang X., Qiao Y., Li W., Zou X., Chen Y., Shen J., Liao Q., Zhang Q., He L., Zhao H. (2018). Human amylin induces CD4+Foxp3+ regulatory T cells in the protection from autoimmune diabetes. Immunol. Res..

[37] Kayser B., Prifti E., Lhomme M., Belda E., Dao M., Aron-Wisnewsky J., Kontush A., Zucker J., Rizkalla S., Dugail I. (2019). Elevated serum ceramides are linked with obesity-associated gut dysbiosis and impaired glucose metabolism. Metabolomics Off. J. Metabolomic Soc..

[38] Nie X., Chen J., Ma X., Ni Y., Shen Y., Yu H., Panagiotou G., Bao Y. (2020). A metagenome-wide association study of gut microbiome and visceral fat accumulation. Comput. Struct. Biotechnol. J..

[39] Bolger A., Lohse M., Usadel B. (2014). Trimmomatic: A flexible trimmer for Illumina sequence data. Bioinformatics.

[40] Segata N., Izard J., Waldron L., Gevers D., Miropolsky L., Garrett W., Huttenhower C. (2011). Metagenomic biomarker discovery and explanation. Genome Biol..

[41] Rodriguez Camargo D., Tripsianes K., Kapp T., Mendes J., Schubert J., Cordes B., Reif B. (2015). Cloning, expression and purification of the human Islet Amyloid Polypeptide (hIAPP) from *Escherichia coli*. Protein Expr. Purif..

[42] Bhattacharya S., Latha J., Kumresan R., Singh S. (2007). Cloning and expression of human islet amyloid polypeptide in cultured cells. Biochem. Biophys. Res. Commun..

[43] Chen W., Wang F., Tian C., Wang Y., Xu S., Wang R., Hou K., Zhao P., Yu L., Lu Z. (2018). Transgenic Silkworm-Based Silk Gland Bioreactor for Large Scale Production of Bioactive Human Platelet-Derived Growth Factor (PDGF-BB) in Silk Cocoons. Int. J. Mol. Sci..

[44] Tao J., Yang M., Wu H., Ma T., He C., Chai M., Zhang X., Zhang J., Ding F., Wang S. (2018). Effects of AANAT overexpression on the inflammatory responses and autophagy activity in the cellular and transgenic animal levels. Autophagy.

[45] Ji M.R., Lee S.I., Jang Y.J., Jeon M.H., Kim J.S., Kim K.W., Park J.K., Yoo J.G., Jeon I.S., Kwon D.J. (2015). STAT5 plays a critical role in regulating the 5’-flanking region of the porcine whey acidic protein gene in transgenic mice. Mol. Reprod. Dev..

[46] Bussmann U.A., Perez Saez J.M., Bussmann L.E., Baranao J.L. (2013). Aryl hydrocarbon receptor activation leads to impairment of estrogen-driven chicken vitellogenin promoter activity in LMH cells. Comp. Biochem. Physiol. C Toxicol. Pharmacol..

[47] Molinero N., Ruiz L., Milani C., Gutiérrez-Díaz I., Sánchez B., Mangifesta M., Segura J., Cambero I., Campelo A., García-Bernardo C. (2019). The human gallbladder microbiome is related to the physiological state and the biliary metabolic profile. Microbiome.

[48] Lai Z., Tseng C., Ho H., Cheung C., Lin J., Chen Y., Cheng F., Hsu Y., Lin J., El-Omar E. (2018). Fecal microbiota transplantation confers beneficial metabolic effects of diet and exercise on diet-induced obese mice. Sci. Rep..

[49] Tun H., Bridgman S., Chari R., Field C., Guttman D., Becker A., Mandhane P., Turvey S., Subbarao P., Sears M. (2018). Roles of Birth Mode and Infant Gut Microbiota in Intergenerational Transmission of Overweight and Obesity from Mother to Offspring. JAMA Pediatr..

[50] Sorbara M., Littmann E., Fontana E., Moody T., Kohout C., Gjonbalaj M., Eaton V., Seok R., Leiner I., Pamer E. (2020). Functional and Genomic Variation between Human-Derived Isolates of Lachnospiraceae Reveals Inter- and Intra-Species Diversity. Cell Host Microbe.

[51] Arias L., Goig G., Cardona P., Torres-Puente M., Díaz J., Rosales Y., Garcia E., Tapia G., Comas I., Vilaplana C. (2019). Influence of Gut Microbiota on Progression to Tuberculosis Generated by High Fat Diet-Induced Obesity in C3HeB/FeJ Mice. Front. Immunol..

